# Twenty years of gender equality research: A scoping review based on a new semantic indicator

**DOI:** 10.1371/journal.pone.0256474

**Published:** 2021-09-21

**Authors:** Paola Belingheri, Filippo Chiarello, Andrea Fronzetti Colladon, Paola Rovelli

**Affiliations:** 1 Dipartimento di Ingegneria dell’Energia, dei Sistemi, del Territorio e delle Costruzioni, Università degli Studi di Pisa, Largo L. Lazzarino, Pisa, Italy; 2 Department of Engineering, University of Perugia, Perugia, Italy; 3 Department of Management, Kozminski University, Warsaw, Poland; 4 Faculty of Economics and Management, Centre for Family Business Management, Free University of Bozen-Bolzano, Bozen-Bolzano, Italy; Politecnico di Torino, ITALY

## Abstract

Gender equality is a major problem that places women at a disadvantage thereby stymieing economic growth and societal advancement. In the last two decades, extensive research has been conducted on gender related issues, studying both their antecedents and consequences. However, existing literature reviews fail to provide a comprehensive and clear picture of what has been studied so far, which could guide scholars in their future research. Our paper offers a scoping review of a large portion of the research that has been published over the last 22 years, on gender equality and related issues, with a specific focus on business and economics studies. Combining innovative methods drawn from both network analysis and text mining, we provide a synthesis of 15,465 scientific articles. We identify 27 main research topics, we measure their relevance from a semantic point of view and the relationships among them, highlighting the importance of each topic in the overall gender discourse. We find that prominent research topics mostly relate to women in the workforce–e.g., concerning compensation, role, education, decision-making and career progression. However, some of them are losing momentum, and some other research trends–for example related to female entrepreneurship, leadership and participation in the board of directors–are on the rise. Besides introducing a novel methodology to review broad literature streams, our paper offers a map of the main gender-research trends and presents the most popular and the emerging themes, as well as their intersections, outlining important avenues for future research.

## Introduction

The persistent gender inequalities that currently exist across the developed and developing world are receiving increasing attention from economists, policymakers, and the general public [e.g., 1–3]. Economic studies have indicated that women’s education and entry into the workforce contributes to social and economic well-being [e.g., 4, 5], while their exclusion from the labor market and from managerial positions has an impact on overall labor productivity and income per capita [6, 7]. The United Nations selected gender equality, with an emphasis on female education, as part of the Millennium Development Goals [8], and gender equality at-large as one of the 17 Sustainable Development Goals (SDGs) to be achieved by 2030 [9]. These latter objectives involve not only developing nations, but rather all countries, to achieve economic, social and environmental well-being.

As is the case with many SDGs, gender equality is still far from being achieved and persists across education, access to opportunities, or presence in decision-making positions [7, 10, 11]. As we enter the last decade for the SDGs’ implementation, and while we are battling a global health pandemic, effective and efficient action becomes paramount to reach this ambitious goal.

Scholars have dedicated a massive effort towards understanding gender equality, its determinants, its consequences for women and society, and the appropriate actions and policies to advance women’s equality. Many topics have been covered, ranging from women’s education and human capital [12, 13] and their role in society [e.g., 14, 15], to their appointment in firms’ top ranked positions [e.g., 16, 17] and performance implications [e.g., 18, 19]. Despite some attempts, extant literature reviews provide a narrow view on these issues, restricted to specific topics–e.g., female students’ presence in STEM fields [20], educational gender inequality [5], the gender pay gap [21], the glass ceiling effect [22], leadership [23], entrepreneurship [24], women’s presence on the board of directors [25, 26], diversity management [27], gender stereotypes in advertisement [28], or specific professions [29]. A comprehensive view on gender-related research, taking stock of key findings and under-studied topics is thus lacking.

Extant literature has also highlighted that gender issues, and their economic and social ramifications, are complex topics that involve a large number of possible antecedents and outcomes [7]. Indeed, gender equality actions are most effective when implemented in unison with other SDGs (e.g., with SDG 8, see [30]) in a synergetic perspective [10]. Many bodies of literature (e.g., business, economics, development studies, sociology and psychology) approach the problem of achieving gender equality from different perspectives–often addressing specific and narrow aspects. This sometimes leads to a lack of clarity about how different issues, circumstances, and solutions may be related in precipitating or mitigating gender inequality or its effects. As the number of papers grows at an increasing pace, this issue is exacerbated and there is a need to step back and survey the body of gender equality literature as a whole. There is also a need to examine synergies between different topics and approaches, as well as gaps in our understanding of how different problems and solutions work together. Considering the important topic of women’s economic and social empowerment, this paper aims to fill this gap by answering the following research question: *what are the most relevant findings in the literature on gender equality and how do they relate to each other*?

To do so, we conduct a scoping review [31], providing a synthesis of 15,465 articles dealing with gender equity related issues published in the last twenty-two years, covering both the periods of the MDGs and the SDGs (i.e., 2000 to mid 2021) in all the journals indexed in the Academic Journal Guide’s 2018 ranking of business and economics journals. Given the huge amount of research conducted on the topic, we adopt an innovative methodology, which relies on social network analysis and text mining. These techniques are increasingly adopted when surveying large bodies of text. Recently, they were applied to perform analysis of online gender communication differences [32] and gender behaviors in online technology communities [33], to identify and classify sexual harassment instances in academia [34], and to evaluate the gender inclusivity of disaster management policies [35].

Applied to the title, abstracts and keywords of the articles in our sample, this methodology allows us to identify a set of 27 recurrent topics within which we automatically classify the papers. Introducing additional novelty, by means of the Semantic Brand Score (SBS) indicator [36] and the SBS BI app [37], we assess the importance of each topic in the overall gender equality discourse and its relationships with the other topics, as well as trends over time, with a more accurate description than that offered by traditional literature reviews relying solely on the number of papers presented in each topic.

This methodology, applied to gender equality research spanning the past twenty-two years, enables two key contributions. First, we extract the main message that each document is conveying and how this is connected to other themes in literature, providing a rich picture of the topics that are at the center of the discourse, as well as of the emerging topics. Second, by examining the semantic relationship between topics and how tightly their discourses are linked, we can identify the key relationships and connections between different topics. This semi-automatic methodology is also highly reproducible with minimum effort.

This literature review is organized as follows. In the next section, we present how we selected relevant papers and how we analyzed them through text mining and social network analysis. We then illustrate the importance of 27 selected research topics, measured by means of the SBS indicator. In the results section, we present an overview of the literature based on the SBS results–followed by an in-depth narrative analysis of the top 10 topics (i.e., those with the highest SBS) and their connections. Subsequently, we highlight a series of under-studied connections between the topics where there is potential for future research. Through this analysis, we build a map of the main gender-research trends in the last twenty-two years–presenting the most popular themes. We conclude by highlighting key areas on which research should focused in the future.

## Methods

Our aim is to map a broad topic, gender equality research, that has been approached through a host of different angles and through different disciplines. Scoping reviews are the most appropriate as they provide the freedom to map different themes and identify literature gaps, thereby guiding the recommendation of new research agendas [38].

Several practical approaches have been proposed to identify and assess the underlying topics of a specific field using big data [39–41], but many of them fail without proper paper retrieval and text preprocessing. This is specifically true for a research field such as the gender-related one, which comprises the work of scholars from different backgrounds. In this section, we illustrate a novel approach for the analysis of scientific (gender-related) papers that relies on methods and tools of social network analysis and text mining. Our procedure has four main steps: (1) data collection, (2) text preprocessing, (3) keywords extraction and classification, and (4) evaluation of semantic importance and image.

### Data collection

In this study, we analyze 22 years of literature on gender-related research. Following established practice for scoping reviews [42], our data collection consisted of two main steps, which we summarize here below.

Firstly, we retrieved from the Scopus database all the articles written in English that contained the term “gender” in their title, abstract or keywords and were published in a journal listed in the Academic Journal Guide 2018 ranking of the Chartered Association of Business Schools (CABS) (https://charteredabs.org/wp-content/uploads/2018/03/AJG2018-Methodology.pdf), considering the time period from Jan 2000 to May 2021. We used this information considering that abstracts, titles and keywords represent the most informative part of a paper, while using the full-text would increase the signal-to-noise ratio for information extraction. Indeed, these textual elements already demonstrated to be reliable sources of information for the task of domain lexicon extraction [43, 44]. We chose Scopus as source of literature because of its popularity, its update rate, and because it offers an API to ease the querying process. Indeed, while it does not allow to retrieve the full text of scientific articles, the Scopus API offers access to titles, abstracts, citation information and metadata for all its indexed scholarly journals. Moreover, we decided to focus on the journals listed in the AJG 2018 ranking because we were interested in reviewing business and economics related gender studies only. The AJG is indeed widely used by universities and business schools as a reference point for journal and research rigor and quality. This first step, executed in June 2021, returned more than 55,000 papers.

In the second step–because a look at the papers showed very sparse results, many of which were not in line with the topic of this literature review (e.g., papers dealing with health care or medical issues, where the word *gender* indicates the gender of the patients)–we applied further inclusion criteria to make the sample more focused on the topic of this literature review (i.e., women’s gender equality issues). Specifically, we only retained those papers mentioning, in their title and/or abstract, both gender-related keywords (e.g., daughter, female, mother) and keywords referring to bias and equality issues (e.g., equality, bias, diversity, inclusion). After text pre-processing (see next section), keywords were first identified from a frequency-weighted list of words found in the titles, abstracts and keywords in the initial list of papers, extracted through text mining (following the same approach as [43]). They were selected by two of the co-authors independently, following respectively a bottom up and a top-down approach. The bottom-up approach consisted of examining the words found in the frequency-weighted list and classifying those related to gender and equality. The top-down approach consisted in searching in the word list for notable gender and equality-related words. Table 1 reports the sets of keywords we considered, together with some examples of words that were used to search for their presence in the dataset (a full list is provided in the S1 Text). At end of this second step, we obtained a final sample of 15,465 relevant papers.

**Table 1:** Examples of keywords used to identify gender-related papers relevant for the aim of the literature review.

	Keyword set	Examples of searched words
Gender	Bride	*bride*
	Daughter	*daughter*, *daughterhood*
	Female	female*, *femaling*
	Femini	*feminist*, *feminism*, *femininity*
	Girl	*girl*
	Lady	*lady*, *ladies*
	Maid	*maid*
	Mother	*mother*, *maternal*, *maternity*
	Queen	*queen*
	Widow	*widow*
	Wife	*wife*, *wives*
	Woman	*woman*, *women*
Equality	Bias	*bias*, *biases*, *biased*
	Diversity	*diversity*, *diverse*
	Empower	*empower*, *empowering*, *empowerment*
	Equality	*inequality*, *equality*, *discrimination*
	Equity	*equity*, *inequity*, *inequities*
	Homeworking	*homeworker*, *householder*, *homemaking*
	Inclusion	*barrier*, *inclusion*, *inclusive*
	Quota	*quota*
	Stereotype	*stereotype*, *stereotyping*, *stereotyped*

### Text processing and keyword extraction

Text preprocessing aims at structuring text into a form that can be analyzed by statistical models. In the present section, we describe the preprocessing steps we applied to paper titles and abstracts, which, as explained below, partially follow a standard text preprocessing pipeline [45]. These activities have been performed using the R package *udpipe* [46].

The first step is n-gram extraction (i.e., a sequence of words from a given text sample) to identify which n-grams are important in the analysis, since domain-specific lexicons are often composed by bi-grams and tri-grams [47]. Multi-word extraction is usually implemented with statistics and linguistic rules, thus using the statistical properties of n-grams or machine learning approaches [48]. However, for the present paper, we used Scopus metadata in order to have a more effective and efficient n-grams collection approach [49]. We used the keywords of each paper in order to tag n-grams with their associated keywords automatically. Using this greedy approach, it was possible to collect all the keywords listed by the authors of the papers. From this list, we extracted only keywords composed by two, three and four words, we removed all the acronyms and rare keywords (i.e., appearing in less than 1% of papers), and we clustered keywords showing a high orthographic similarity–measured using a Levenshtein distance [50] lower than 2, considering these groups of keywords as representing same concepts, but expressed with different spelling. After tagging the n-grams in the abstracts, we followed a common data preparation pipeline that consists of the following steps: (i) tokenization, that splits the text into tokens (i.e., single words and previously tagged multi-words); (ii) removal of stop-words (i.e. those words that add little meaning to the text, usually being very common and short functional words–such as “and”, “or”, or “of”); (iii) parts-of-speech tagging, that is providing information concerning the morphological role of a word and its morphosyntactic context (e.g., if the token is a determiner, the next token is a noun or an adjective with very high confidence, [51]); and (iv) lemmatization, which consists in substituting each word with its dictionary form (or lemma). The output of the latter step allows grouping together the inflected forms of a word. For example, the verbs “am”, “are”, and “is” have the shared lemma “be”, or the nouns “cat” and “cats” both share the lemma “cat”. We preferred lemmatization over stemming [52] in order to obtain more interpretable results.

In addition, we identified a further set of keywords (with respect to those listed in the “keywords” field) by applying a series of automatic words unification and removal steps, as suggested in past research [53, 54]. We removed: sparse terms (i.e., occurring in less than 0.1% of all documents), common terms (i.e., occurring in more than 10% of all documents) and retained only nouns and adjectives. It is relevant to notice that no document was lost due to these steps. We then used the TF-IDF function [55] to produce a new list of keywords. We additionally tested other approaches for the identification and clustering of keywords–such as TextRank [56] or Latent Dirichlet Allocation [57]–without obtaining more informative results.

### Classification of research topics

To guide the literature analysis, two experts met regularly to examine the sample of collected papers and to identify the main topics and trends in gender research. Initially, they conducted brainstorming sessions on the topics they expected to find, due to their knowledge of the literature. This led to an initial list of topics. Subsequently, the experts worked independently, also supported by the keywords in paper titles and abstracts extracted with the procedure described above.

Considering all this information, each expert identified and clustered relevant keywords into topics. At the end of the process, the two assignments were compared and exhibited a 92% agreement. Another meeting was held to discuss discordant cases and reach a consensus. This resulted in a list of 27 topics, briefly introduced in Table 2 and subsequently detailed in the following sections.

**Table 2:** List of selected topics.

Topic	Short Description
Behavior	Behavioral aspects related to gender
Board of directors	Women in boards of directors
Career Progression	Women’s promotion and career advancement
Compensation	Salary and rewards in relation to employment
Culture	Ideas, customs and social behaviors, including bias and stereotypes
Decision-making	The decision-making process
Education	Primary, secondary and tertiary education
Empowerment	Authority, power and self-confidence
Entrepreneurship	Women starting their own enterprises
Family	Women’s relationship with family and family obligations, wok-life balance
Feminine	Female characteristics
Governance	The governance structures of firms and society
Hiring	Appointing women to positions within the workforce
Human Capital	The intellectual capital resulting from education and social capital
Leadership	Leadership skills and leadership positions
Management	Managerial practices and processes
Masculine	Male characteristics
Network	Networking dynamics as they relate to women
Organization	The organization of firms
Parenting	The act of raising children and its implications
Performance	Measuring the work output of individuals, teams and organizations
Personality	Traits and individual characteristics of women
Politics	Policies and regulations, women in politics
Reputation	How women are viewed by their colleagues, peers and society
Role	The roles covered by women in the workforce
Sustainability	Women’s relation to sustainability and social responsibility
Well-Being	Psychological, personal, and social welfare of women

### Evaluation of semantic importance

Working on the lemmatized corpus of the 15,465 papers included in our sample, we proceeded with the evaluation of semantic importance trends for each topic and with the analysis of their connections and prevalent textual associations. To this aim, we used the Semantic Brand Score indicator [36], calculated through the SBS BI webapp [37] that also produced a brand image report for each topic. For this study we relied on the computing resources of the ENEA/CRESCO infrastructure [58].

The Semantic Brand Score (SBS) is a measure of semantic importance that combines methods of social network analysis and text mining. It is usually applied for the analysis of (big) textual data to evaluate the importance of one or more brands, names, words, or sets of keywords [36]. Indeed, the concept of “brand” is intended in a flexible way and goes beyond products or commercial brands. In this study, we evaluate the SBS time-trends of the keywords defining the research topics discussed in the previous section. Semantic importance comprises the three dimensions of topic prevalence, diversity and connectivity. Prevalence measures how frequently a research topic is used in the discourse. The more a topic is mentioned by scientific articles, the more the research community will be aware of it, with possible increase of future studies; this construct is partly related to that of brand awareness [59]. This effect is even stronger, considering that we are analyzing the title, abstract and keywords of the papers, i.e. the parts that have the highest visibility. A very important characteristic of the SBS is that it considers the relationships among words in a text. Topic importance is not just a matter of how frequently a topic is mentioned, but also of the associations a topic has in the text. Specifically, texts are transformed into networks of co-occurring words, and relationships are studied through social network analysis [60]. This step is necessary to calculate the other two dimensions of our semantic importance indicator. Accordingly, a social network of words is generated for each time period considered in the analysis–i.e., a graph made of *n* nodes (words) and *E* edges weighted by co-occurrence frequency, with *W* being the set of edge weights. The keywords representing each topic were clustered into single nodes.

The construct of diversity relates to that of brand image [59], in the sense that it considers the richness and distinctiveness of textual (topic) associations. Considering the above-mentioned networks, we calculated diversity using the *distinctiveness centrality* metric–as in the formula presented by Fronzetti Colladon and Naldi [61].

Lastly, connectivity was measured as the *weighted betweenness centrality* [62, 63] of each research topic node. We used the formula presented by Wasserman and Faust [60]. The dimension of connectivity represents the “brokerage power” of each research topic–i.e., how much it can serve as a bridge to connect other terms (and ultimately topics) in the discourse [36].

The SBS is the final composite indicator obtained by summing the standardized scores of prevalence, diversity and connectivity. Standardization was carried out considering all the words in the corpus, for each specific timeframe.

This methodology, applied to a large and heterogeneous body of text, enables to automatically identify two important sets of information that add value to the literature review. Firstly, the relevance of each topic in literature is measured through a composite indicator of semantic importance, rather than simply looking at word frequencies. This provides a much richer picture of the topics that are at the center of the discourse, as well as of the topics that are emerging in the literature. Secondly, it enables to examine the extent of the semantic relationship between topics, looking at how tightly their discourses are linked. In a field such as gender equality, where many topics are closely linked to each other and present overlaps in issues and solutions, this methodology offers a novel perspective with respect to traditional literature reviews. In addition, it ensures reproducibility over time and the possibility to semi-automatically update the analysis, as new papers become available.

## Results

### Overview of main topics

In terms of descriptive textual statistics, our corpus is made of 15,465 text documents, consisting of a total of 2,685,893 lemmatized tokens (words) and 32,279 types. As a result, the type-token ratio is 1.2%. The number of hapaxes is 12,141, with a hapax-token ratio of 37.61%.

**Fig 1:**
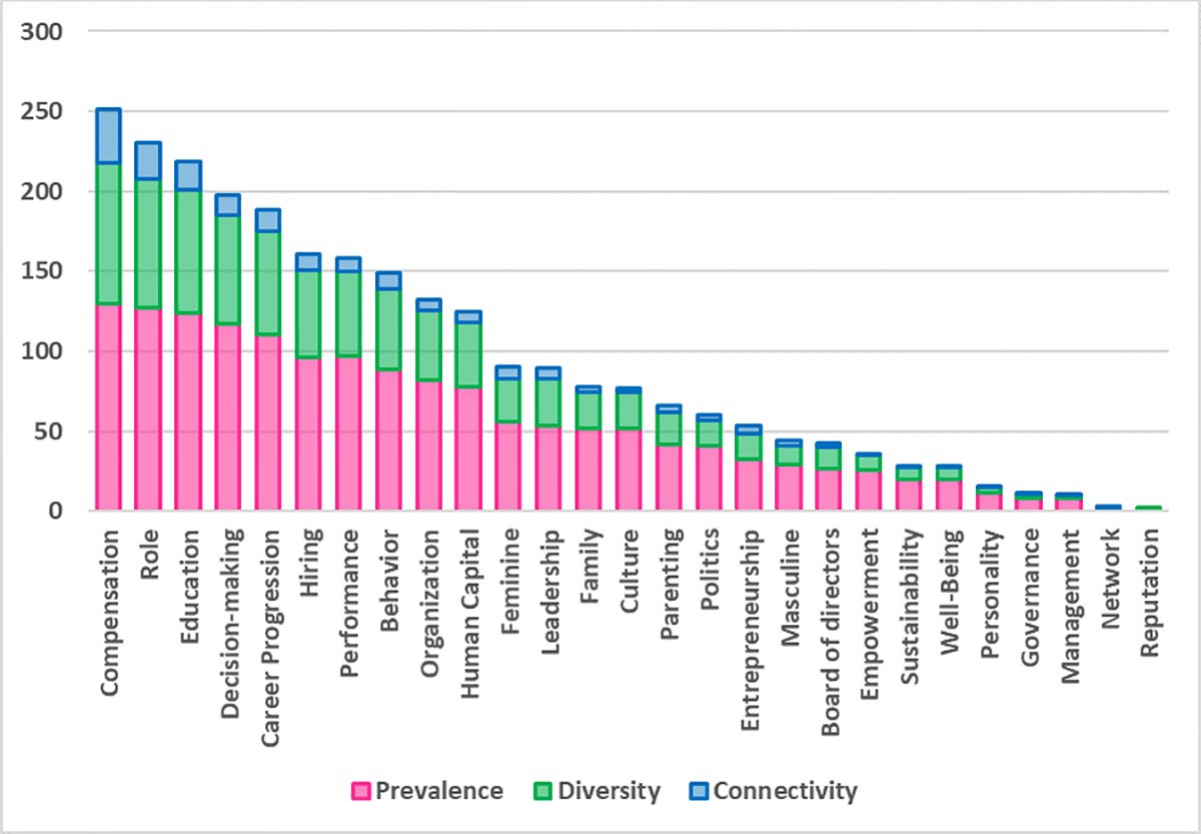
Topic importance (SBS).

Fig 1 shows the list of 27 topics by decreasing SBS. The most researched topic is *compensation*, exceeding all others in prevalence, diversity, and connectivity. This means it is not only mentioned more often than other topics, but it is also connected to a greater number of other topics and is central to the discourse on gender equality. The next four topics are, in order of SBS, *role*, *education*, *decision-making*, and *career progression*. These topics, except for *education*, all concern women in the workforce. Between these first five topics and the following ones there is a clear drop in SBS scores. In particular, the topics that follow have a lower connectivity than the first five. They are *hiring*, *performance*, *behavior*, *organization*, and *human capital*. Again, except for *behavior* and *human capital*, the other three topics are purely related to women in the workforce. After another drop-off, the following topics deal prevalently with women in society. This trend highlights that research on gender in business journals has so far mainly paid attention to the conditions that women experience in business contexts, while also devoting some attention to women in society.

**Fig 2:**
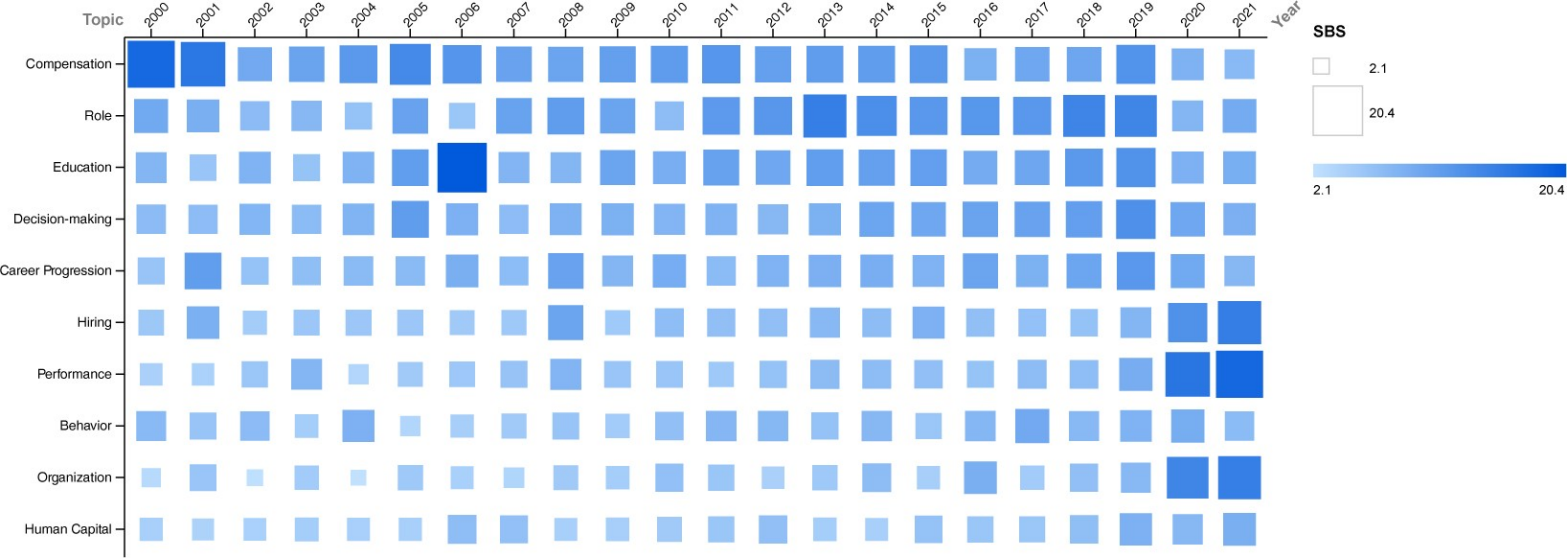
SBS time series of the top 10 topics.

Fig 2 shows the SBS time series of the top 10 topics. While there has been a general increase in the number of Scopus-indexed publications in the last decade, we notice that some SBS trends remain steady, or even decrease. In particular, we observe that the main topic of the last twenty-two years, *compensation*, is losing momentum. Since 2016, it has been surpassed by *decision-making*, *education* and *role*, which may indicate that literature is increasingly attempting to identify root causes of compensation inequalities. Moreover, in the last two years, the topics of *hiring*, *performance*, and *organization* are experiencing the largest importance increase.

**Fig 3:**
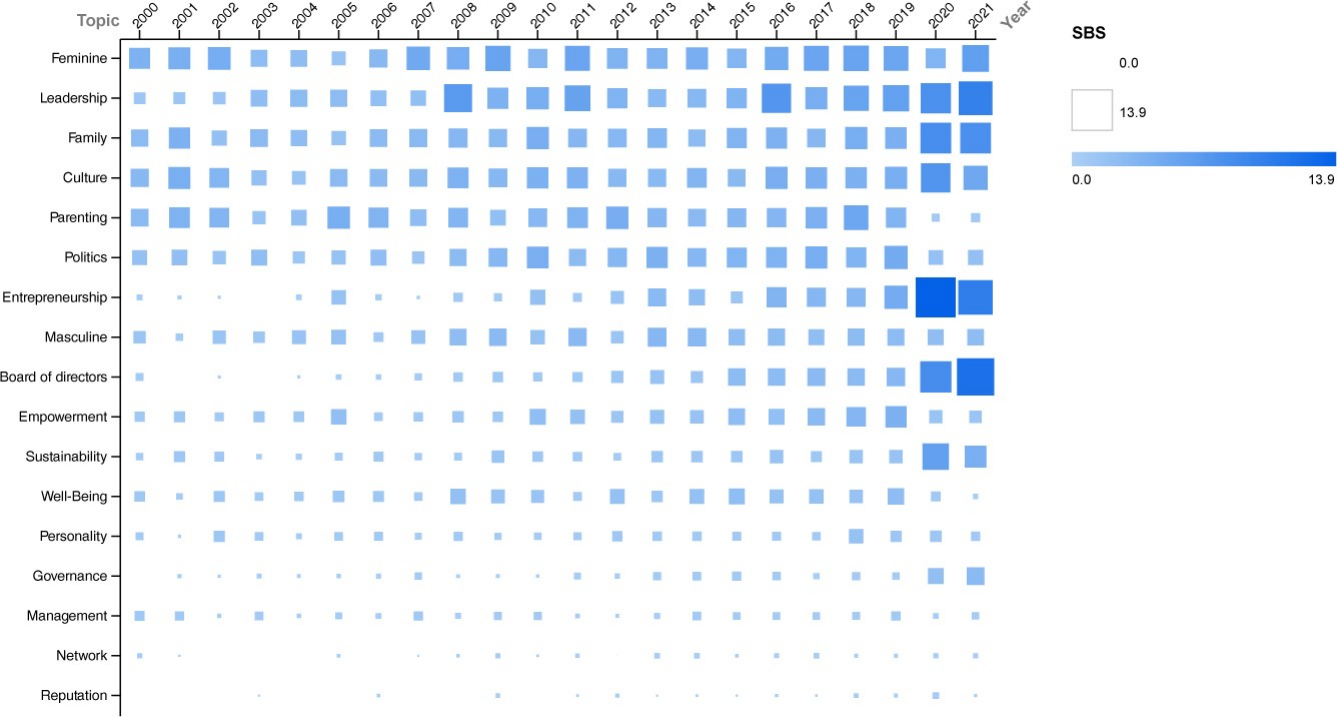
SBS time series of the other topics.

Fig 3 shows the SBS time trends of the remaining 17 topics (i.e., those not in the top 10). As we can see from the graph, there are some that maintain a steady trend–such as *reputation*, *management*, *networks* and *governance*, which also seem to have little importance. More relevant topics with average stationary trends (except for the last two years) are *culture*, *family*, and *parenting*. The *feminine* topic is among the most important here, and one of those that exhibit the larger variations over time (similarly to *leadership*). On the other hand, the are some topics that, even if not among the most important, show increasing SBS trends; therefore, they could be considered as emerging topics and could become popular in the near future. These are *entrepreneurship*, *leadership*, *board of directors*, and *sustainability*. These emerging topics are also interesting to anticipate future trends in gender equality research that are conducive to overall equality in society.

**Fig 4:**
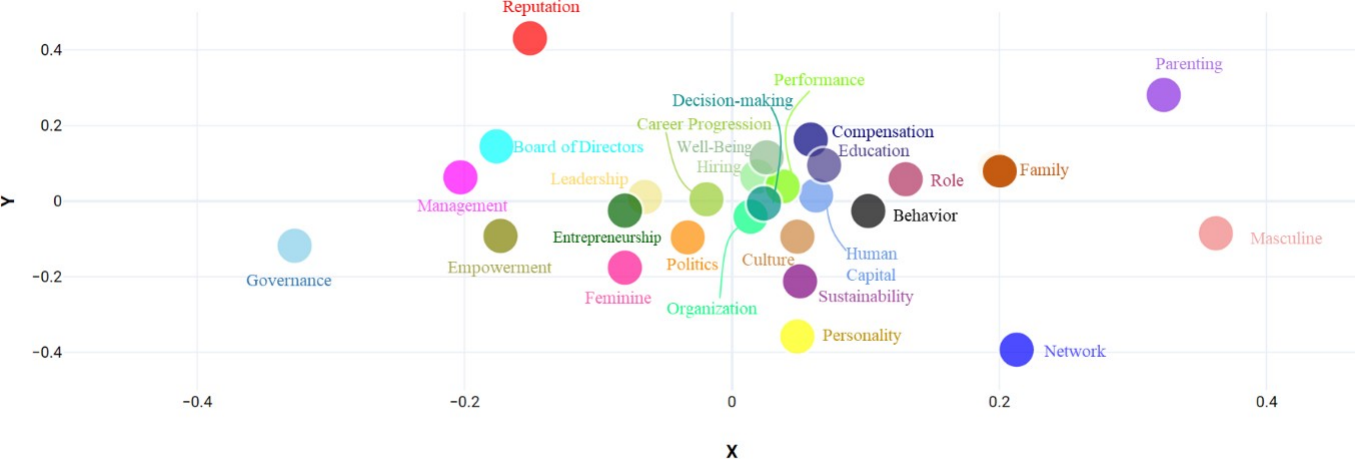
Topic similarity.

In addition to the SBS score of the different topics, the network of terms they are associated to enables to gauge the extent to which their images (textual associations) overlap or differ (Fig 4).

There is a central cluster of topics with high similarity, which are all connected with women in the workforce. The cluster includes topics such as *organization*, *decision-making*, *performance*, *hiring*, *human capital*, *education* and *compensation*. In addition, the topic of *well-being* is found within this cluster, suggesting that women’s equality in the workforce is associated to well-being considerations. The emerging topics of *entrepreneurship* and *leadership* are also closely connected with each other, possibly implying that leadership is a much-researched quality in female entrepreneurship. Topics that are relatively more distant include *personality*, *politics*, *feminine*, *empowerment*, *management*, *board of directors*, *reputation*, *governance*, *parenting*, *masculine* and *network*.

The following sections describe the top 10 topics and their main associations in literature (see Table 3), while providing a brief overview of the emerging topics.

**Table 3:** Top associations.

Topic	Top associations (other topics in bold)
Behavior	social, work, **Role**, differences, related, **Decision-making**, child, positive, group, individual, self, influence, relationship, stereotype, health, inequality, change, **Education**, student, participant, **Career Progression**, **Human Capital**, experience, **Performance**, **Parenting**, intention
Career Progression	**Role**, inequality, difference**, Decision-making**, work, social, equity, **Hiring**, **Education**, **Organization**, **Performance**, level, **Compensation**, development, policy, examine, role, self, experience, **Leadership**, support, **Human Capital**, individual, **Family**, perceive, academic, differences
Compensation	gap, **Role**, difference, inequality, **Hiring**, **Education**, work, increase, higher, lower, market, less, labor, household, low, **Career Progression**, age, time, high, labour, attention, discrimination, change, country, individual, status
Decision Making	**Career Progression**, **Performance**, social, work, **Role**, **Organization**, inequality, household, group, policy, **Human Capital**, process, **Behavior**, health, **Hiring**, level, role, individual, **Compensation**, **Education**, equity, **Leadership**, stereotype, different, **Politics**, change
Education	age, inequality, level, **Compensation**, study, social, health, gap, status, equity, student, **Human Capital**, **Career Progression**, child, **Hiring**, school, economic, policy, work, **Parenting**, experience, higher, access, household, **Decision-making,** development
Hiring	**Role**, work, **Career Progression**, **Education**, discrimination, level, **Decision-making**, time, **Human Capital**, gap, sector, **Performance**, market, social, increase, status, **Organization**, policy, inequality, experience, differences, lower, equity, high, data, satisfaction,
Human Capital	**Education**, **Role**, work, **Decision-making**, social, **Hiring**, **Career Progression**, **Performance**, self, **Behavior**, health, **Compensation**, **Leadership**, student, **Organization**, group, child, individual, development, age, differences, lack, gap, focus, change
Organization	work, **Role**, **Career Progression**, inequality, **Decision-making**, **Leadership**, social, diversity, policy, level, change, **Performance**, employee, individual, **Hiring**, equity, **Culture**, practice, value, **Human Capital**, management, structure, discrimination, **Behavior**, **Governance**
Performance	**Role**, **Decision-making**, **Career Progression**, stereotype, work, **Compensation**, **Hiring**, **Organization**, **Human Capital**, self, impact, social, **Education**, **Behavior**, difference, high, firm, threat, student, inequality, role, **Board of directors**, increase, relationship, experience
Role	**Compensation**, **Career Progression**, work, **Hiring**, **Performance**, **Organization**, firm, **Board of directors**, **Behavior**, social, **Decision-making**, role, **Human Capital**, employee, less, increase, experience, traditional, **Leadership**, stereotype, sector, **Entrepreneurship**, business, gap, group, data

#### Compensation

The topic of compensation is related to the topics of *role*, *hiring*, *education* and *career progression*, however, also sees a very high association with the words *gap* and *inequality*. Indeed, a well-known debate in degrowth economics centers around whether and how to adequately *compensate* women for their childbearing, childrearing, caregiver and household work [e.g., 30].

Even in paid work, women continue being offered lower *compensations* than their male counterparts who have the same job or cover the same role [64–67]. This severe inequality has been widely studied by scholars over the last twenty-two years. Dealing with this topic, some specific *roles* have been addressed. Specifically, research highlighted differences in compensation between female and male CEOs [e.g., 68], top executives [e.g., 69], and boards’ directors [e.g., 70]. Scholars investigated the determinants of these gaps, such as the gender composition of the board [e.g., 71–73] or women’s individual characteristics [e.g., 71, 74].

Among these individual characteristics, *education* plays a relevant role [75]. Education is indeed presented as the solution for women, not only to achieve top executive roles, but also to reduce wage inequality [e.g., 76, 77]. Past research has highlighted education influences on gender wage gaps, specifically referring to gender differences in skills [e.g., 78], college majors [e.g., 79], and college selectivity [e.g., 80].

Finally, the wage gap issue is strictly interrelated with *hiring*–e.g., looking at whether being a mother affects hiring and compensation [e.g., 65, 81] or relating compensation to unemployment [e.g., 82]–and *career progression*–for instance looking at meritocracy [83, 84] or the characteristics of the boss for whom women work [e.g., 85].

#### Role

The *roles* covered by women have been deeply investigated. Scholars have focused on the role of women in their families and the society as a whole [e.g., 14, 15], and, more widely, in business contexts [e.g., 18, 81]. Indeed, despite still lagging behind their male counterparts [e.g., 86, 87], in the last decade there has been an increase in top ranked positions achieved by women [e.g., 88, 89]. Following this phenomenon, scholars have posed greater attention towards the presence of women in the *board of directors* [e.g., 16, 18, 90, 91], given the increasing pressure to appoint female directors that firms, especially listed ones, have experienced. Other scholars have focused on the presence of women covering the role of CEO [e.g., 17, 92] or being part of the top management team [e.g., 93]. Irrespectively of the level of analysis, all these studies tried to uncover the antecedents of women’s presence among top managers [e.g., 92, 94] and the consequences of having a them involved in the firm’s *decision-making*–e.g., on *performance* [e.g., 19, 95, 96], risk [e.g., 97, 98], and corporate social responsibility [e.g., 99, 100].

Besides studying the difficulties and discriminations faced by women in getting a job [81, 101], and, more specifically in the *hiring*, appointment, or *career progression* to these apical roles [e.g., 70, 83], the majority of research of women’s roles dealt with *compensation* issues. Specifically, scholars highlight the pay-gap that still exists between women and men, both in general [e.g., 64, 65], as well as referring to boards’ directors [e.g., 70, 102], CEOs and executives [e.g., 69, 103, 104].

Finally, other scholars focused on the behavior of women when dealing with business. In this sense, particular attention has been paid to *leadership* and entrepreneurial behaviors. The former quite overlaps with dealing with the roles mentioned above, but also includes aspects such as leaders being stereotyped as masculine [e.g., 105], the need for greater exposure to female leaders to reduce biases [e.g., 106], or female leaders acting as queen bees [e.g., 107]. Regarding *entrepreneurship*, scholars mainly investigated women’s entrepreneurial entry [e.g., 108, 109], differences between female and male entrepreneurs in the evaluations and funding received from investors [e.g., 110, 111], and their performance gap [e.g., 112, 113].

#### Education

*Education* has long been recognized as key to social advancement and economic stability [114], for job progression and also a barrier to gender equality, especially in STEM-related fields. Research on education and gender equality is mostly linked with the topics of *compensation*, *human capital*, *career progression*, *hiring*, *parenting* and *decision-making*.

Education contributes to a higher *human capital* [115] and constitutes an investment on the part of women towards their future. In this context, literature points to the gender gap in educational attainment, and the consequences for women from a social, economic, personal and professional standpoint. Women are found to have less access to formal education and information, especially in emerging countries, which in turn may cause them to lose social and economic opportunities [e.g., 12, 116–119]. Education in local and rural communities is also paramount to communicate the benefits of female *empowerment*, contributing to overall societal well-being [e.g., 120].

Once women access education, the image they have of the world and their place in society (i.e., habitus) affects their education performance [13] and is passed on to their children. These situations reinforce gender stereotypes, which become self-fulfilling prophecies that may negatively affect female students’ performance by lowering their confidence and heightening their anxiety [121, 122]. Besides formal education, also the information that women are exposed to on a daily basis contributes to their *human capital*. Digital inequalities, for instance, stems from men spending more time online and acquiring higher digital skills than women [123].

Education is also a factor that should boost employability of candidates and thus *hiring*, *career progression* and *compensation*, however the relationship between these factors is not straightforward [115]. First, educational choices (*decision-making*) are influenced by variables such as self-efficacy and the presence of barriers, irrespectively of the career opportunities they offer, especially in STEM [124]. This brings additional difficulties to women’s enrollment and persistence in scientific and technical fields of study due to stereotypes and biases [125, 126]. Moreover, access to education does not automatically translate into job opportunities for women and minority groups [127, 128] or into female access to managerial positions [129].

Finally, *parenting* is reported as an antecedent of education [e.g., 130], with much of the literature focusing on the role of parents’ education on the opportunities afforded to children to enroll in education [131–134] and the role of parenting in their offspring’s perception of study fields and attitudes towards learning [135–138]. Parental education is also a predictor of the other related topics, namely *human capital* and *compensation* [139].

#### Decision-making

This literature mainly points to the fact that women are thought to make decisions differently than men. Women have indeed different priorities, such as they care more about people’s well-being, working with people or helping others, rather than maximizing their personal (or their firm’s) gain [140]. In other words, women typically present more communal than agentic behaviors, which are instead more frequent among men [141]. These different attitude, behavior and preferences in turn affect the decisions they make [e.g., 142] and the decision-making of the firm in which they work [e.g., 143].

At the individual level, gender affects, for instance, *career* aspirations [e.g., 144] and choices [e.g., 142, 145], or the decision of creating a venture [e.g., 108, 109, 146]. Moreover, in everyday life, women and men make different decisions regarding partners [e.g., 147], childcare [e.g., 148], education [e.g., 149], attention to the environment [e.g., 150] and politics [e.g., 151].

At the firm level, scholars highlighted, for example, how the presence of women in the board affects corporate decisions [e.g., 152, 153], that female CEOs are more conservative in accounting decisions [e.g., 154], or that female CFOs tend to make more conservative decisions regarding the firm’s financial reporting [e.g., 155]. Nevertheless, firm level research also investigated decisions that, influenced by gender bias, affect women, such as those pertaining *hiring* [e.g., 156, 157], *compensation* [e.g., 73, 158], or the empowerment of women once appointed [159].

#### Career progression

Once women have entered the workforce, the key aspect to achieve gender equality becomes *career progression*, including efforts toward overcoming the glass ceiling. Indeed, according to the SBS analysis, career progression is highly related to words such as work, social issues and equality. The topic with which it has the highest semantic overlap is *role*, followed by *decision-making*, *hiring*, *education*, *compensation*, *leadership*, *human capital*, and *family*.

Career progression implies an advancement in the hierarchical ladder of the firm, assigning managerial roles to women. Coherently, much of the literature has focused on identifying rationales for a greater female participation in the top management team and board of directors [e.g., 95] as well as the best criteria to ensure that the *decision-makers* promote the most valuable employees irrespectively of their individual characteristics, such as gender [e.g., 84]. The link between *career progression*, *role* and *compensation* is often provided in practice by performance appraisal exercises, frequently rooted in a culture of meritocracy that guides bonuses, salary increases and promotions. However, performance appraisals can actually mask gender-biased decisions where women are held to higher standards than their male colleagues [e.g., 83, 84, 95, 160, 161]. Women often have less opportunities to gain *leadership* experience and are less visible than their male colleagues, which constitute barriers to career advancement [e.g., 162]. Therefore, transparency and accountability, together with procedures that discourage discretionary choices, are paramount to achieve a fair career progression [e.g., 84], together with the relaxation of strict job boundaries in favor of cross-functional and self-directed tasks [e.g., 163].

In addition, a series of stereotypes about the type of *leadership* characteristics that are required for top management positions, which fit better with typical male and agentic attributes, are another key barrier to career advancement for women [e.g., 92, 160].

#### Hiring

*Hiring* is the entrance gateway for women into the workforce. Therefore, it is related to other workforce topics such as *compensation*, *role*, *career progression*, *decision-making*, *human capital*, *performance*, *organization* and *education*.

A first stream of literature focuses on the process leading up to candidates’ job applications, demonstrating that bias exists before positions are even opened, and it is perpetuated both by men and women through networking and gatekeeping practices [e.g., 164, 165].

The hiring process itself is also subject to biases [166], for example gender-congruity bias that leads to men being preferred candidates in male-dominated sectors [e.g., 167], women being hired in positions with higher risk of failure [e.g., 168] and limited transparency and accountability afforded by written processes and procedures [e.g., 164] that all contribute to ascriptive inequality. In addition, providing incentives for evaluators to hire women may actually work to this end; however, this is not the case when supporting female candidates endangers higher-ranking male ones [169].

Another interesting perspective, instead, looks at top management teams’ composition and the effects on hiring practices, indicating that firms with more women in top management are less likely to lay off staff [e.g., 152].

#### Performance

Several scholars posed their attention towards women’s performance, its consequences [e.g., 170, 171] and the implications of having women in decision-making positions [e.g., 18, 19].

At the individual level, research focused on differences in *educational* and academic performance between women and men, especially referring to the gender gap in STEM fields [e.g., 171]. The presence of stereotype threats–that is the expectation that the members of a social group (e.g., women) “must deal with the possibility of being judged or treated stereotypically, or of doing something that would confirm the stereotype” [172]–affects women’s interested in STEM [e.g., 173], as well as their cognitive ability tests, penalizing them [e.g., 174]. A stronger gender identification enhances this gap [e.g., 175], whereas mentoring and role models can be used as solutions to this problem [e.g., 121]. Despite the negative effect of stereotype threats on girls’ performance [176], female and male students perform equally in mathematics and related subjects [e.g., 177]. Moreover, while individuals’ performance at school and university generally affects their achievements and the field in which they end up working, evidence reveals that performance in math or other scientific subjects does not explain why fewer women enter STEM working fields; rather this gap depends on other aspects, such as culture, past working experiences, or self-efficacy [e.g., 170]. Finally, scholars have highlighted the penalization that women face for their positive performance, for instance when they succeed in traditionally male areas [e.g., 178]. This penalization is explained by the violation of gender-stereotypic prescriptions [e.g., 179, 180], that is having women well performing in agentic areas, which are typical associated to men. Performance penalization can thus be overcome by clearly conveying communal characteristics and behaviors [178].

Evidence has been provided on how the involvement of women in *boards of directors* and *decision-making* positions affects firms’ performance. Nevertheless, results are mixed, with some studies showing positive effects on financial [19, 181, 182] and corporate social performance [99, 182, 183]. Other studies maintain a negative association [e.g., 18], and other again mixed [e.g., 184] or non-significant association [e.g., 185]. Also with respect to the presence of a female CEO, mixed results emerged so far, with some researches demonstrating a positive effect on firm’s performance [e.g., 96, 186], while other obtaining only a limited evidence of this relationship [e.g., 103] or a negative one [e.g., 187].

Finally, some studies have investigated whether and how women’s performance affects their *hiring* [e.g., 101] and *career progression* [e.g., 83, 160]. For instance, academic performance leads to different returns in hiring for women and men. Specifically, high-achieving men are called back significantly more often than high-achieving women, which are penalized when they have a major in mathematics; this result depends on employers’ gendered standards for applicants [e.g., 101]. Once appointed, performance ratings are more strongly related to promotions for women than men, and promoted women typically show higher past performance ratings than those of promoted men. This suggesting that women are subject to stricter standards for promotion [e.g., 160].

#### Behavior

Behavioral aspects related to gender follow two main streams of literature. The first examines female personality and behavior in the workplace, and their alignment with cultural expectations or stereotypes [e.g., 188] as well as their impacts on equality. There is a common bias that depicts women as less agentic than males. Certain characteristics, such as those more congruent with male behaviors–e.g., self-promotion [e.g., 189], negotiation skills [e.g., 190] and general agentic behavior [e.g., 191]–, are less accepted in women. However, characteristics such as individualism in women have been found to promote greater gender equality in society [192]. In addition, behaviors such as display of emotions [e.g., 193], which are stereotypically female, work against women’s acceptance in the workplace, requiring women to carefully moderate their behavior to avoid exclusion. A counter-intuitive result is that women and minorities, which are more marginalized in the workplace, tend to be better problem-solvers in innovation competitions due to their different knowledge bases [194].

The other side of the coin is examined in a parallel literature stream on behavior towards women in the workplace. As a result of biases, prejudices and stereotypes, women may experience adverse behavior from their colleagues, such as incivility and harassment, which undermine their well-being [e.g., 195, 196]. Biases that go beyond gender, such as for overweight people, are also more strongly applied to women [197].

#### Organization

The role of women and gender bias in *organizations* has been studied from different perspectives, which mirror those presented in detail in the following sections. Specifically, most research highlighted the stereotypical view of *leaders* [e.g., 105] and the *roles* played by women within firms, for instance referring to presence in the board of directors [e.g., 18, 90, 91], appointment as CEOs [e.g., 16], or top executives [e.g., 93].

Scholars have investigated antecedents and consequences of the presence of women in these apical roles. On the one side they looked at *hiring* and *career progression* [e.g., 83, 92, 160, 168, 198], finding women typically disadvantaged with respect to their male counterparts. On the other side, they studied women’s *leadership* styles and influence on the firm’s *decision-making* [e.g., 152, 154, 155, 199], with implications for *performance* [e.g., 18, 19, 96].

#### Human capital

*Human capital* is a transverse topic that touches upon many different aspects of female gender equality. As such, it has the most associations with other topics, starting with *education* as mentioned above, with career-related topics such as *role*, *decision-making*, *hiring*, *career progression*, *performance*, *compensation*, *leadership* and *organization*. Another topic with which there is a close connection is *behavior*. In general, human capital is approached both from the education standpoint but also from the perspective of social capital.

The behavioral aspect in human capital comprises research related to gender differences for example in cultural and religious beliefs that influence women’s attitudes and perceptions towards STEM subjects [142, 200–202], towards employment [203] or towards environmental issues [150, 204]. These cultural differences also emerge in the context of globalization which may accelerate gender equality in the workforce [205, 206]. Gender differences also appear in behaviors such as motivation [207], and in negotiation [190], and have repercussions on women’s *decision-making* related to their careers. The so-called gender equality paradox sees women in countries with lower gender equality more likely to pursue studies and careers in STEM fields, whereas the gap in STEM enrollment widens as countries achieve greater equality in society [171].

*Career progression* is modeled by literature as a choice-process where personal preferences, culture and decision-making affect the chosen path and the outcomes. Some literature highlights how women tend to self-select into different professions than men, often due to stereotypes rather than actual ability to perform in these professions [142, 144]. These stereotypes also affect the perceptions of female *performance* or the amount of human capital required to equal male *performance* [110, 193, 208], particularly for mothers [81]. It is therefore often assumed that women are better suited to less visible and less *leadership*-oriented *roles* [209]. Women also express differing preferences towards work-family balance, which affect whether and how they pursue human capital gains [210], and ultimately their *career progression* and *salary*.

On the other hand, men are often unaware of gendered processes and behaviors that they carry forward in their interactions and *decision-making* [211, 212]. Therefore, initiatives aimed at increasing managers’ *human capital*–by raising awareness of gender disparities in their organizations and engaging them in diversity promotion–are essential steps to counter gender bias and segregation [213].

### Emerging topics: Leadership and entrepreneurship

Among the emerging topics, the most pervasive one is women reaching *leadership* positions in the workforce and in society. This is still a rare occurrence for two main types of factors, on the one hand, bias and discrimination make it harder for women to access leadership positions [e.g., 214–216], on the other hand, the competitive nature and high pressure associated with leadership positions, coupled with the lack of women currently represented, reduce women’s desire to achieve them [e.g., 209, 217]. Women are more effective leaders when they have access to education, resources and a diverse environment with representation [e.g., 218, 219].

One sector where there is potential for women to carve out a leadership role is *entrepreneurship*. Although at the start of the millennium the discourse on entrepreneurship was found to be “discriminatory, gender-biased, ethnocentrically determined and ideologically controlled” [220], an increasing body of literature is studying how to stimulate female entrepreneurship as an alternative pathway to wealth, leadership and empowerment [e.g., 221]. Many barriers exist for women to access entrepreneurship, including the institutional and legal environment, social and cultural factors, access to knowledge and resources, and individual behavior [e.g., 222, 223]. Education has been found to raise women’s entrepreneurial intentions [e.g., 224], although this effect is smaller than for men [e.g., 109]. In addition, increasing self-efficacy and risk-taking behavior constitute important success factors [e.g., 225].

Finally, the topic of *sustainability* is worth mentioning, as it is the primary objective of the SDGs and is closely associated with societal well-being. As society grapples with the effects of climate change and increasing depletion of natural resources, a narrative has emerged on women and their greater link to the environment [226]. Studies in developed countries have found some support for women leaders’ attention to sustainability issues in firms [e.g., 227–229], and smaller resource consumption by women [230]. At the same time, women will likely be more affected by the consequences of climate change [e.g., 230] but often lack the decision-making power to influence local decision-making on resource management and environmental policies [e.g., 231].

## Research gaps and conclusions

Research on gender equality has advanced rapidly in the past decades, with a steady increase in publications, both in mainstream topics related to women in education and the workforce, and in emerging topics. Through a novel approach combining methods of text mining and social network analysis, we examined a comprehensive body of literature comprising 15,465 papers published between 2000 and mid 2021 on topics related to gender equality. We identified a set of 27 topics addressed by the literature and examined their connections.

At the highest level of abstraction, it is worth noting that papers abound on the identification of issues related to gender inequalities and imbalances in the workforce and in society. Literature has thoroughly examined the (unconscious) biases, barriers, stereotypes, and discriminatory behaviors that women are facing as a result of their gender. Instead, there are much fewer papers that discuss or demonstrate effective solutions to overcome gender bias [e.g., 121, 143, 145, 163, 194, 213, 232]. This is partly due to the relative ease in studying the status quo, as opposed to studying *changes* in the status quo. However, we observed a shift in the more recent years towards solution seeking in this domain, which we strongly encourage future researchers to focus on. In the future, we may focus on collecting and mapping pro-active contributions to gender studies, using additional Natural Language Processing techniques, able to measure the sentiment of scientific papers [43].

All of the mainstream topics identified in our literature review are closely related, and there is a wealth of insights looking at the intersection between issues such as *education* and *career progression* or *human capital* and *role*. However, emerging topics are worthy of being furtherly explored. It would be interesting to see more work on the topic of *female entrepreneurship*, exploring aspects such as *education*, *personality*, *governance*, *management* and *leadership*. For instance, how can education support female entrepreneurship? How can self-efficacy and risk-taking behaviors be taught or enhanced? What are the differences in managerial and governance styles of female entrepreneurs? Which personality traits are associated with successful entrepreneurs? Which traits are preferred by venture capitalists and funding bodies?

The emerging topic of *sustainability* also deserves further attention, as our society struggles with climate change and its consequences. It would be interesting to see more research on the intersection between *sustainability* and *entrepreneurship*, looking at how female entrepreneurs are tackling sustainability issues, examining both their business models and their company *governance*. In addition, scholars are suggested to dig deeper into the relationship between family values and behaviors.

Moreover, it would be relevant to understand how women’s networks (social capital), or the composition and structure of social networks involving both women and men, enable them to increase their remuneration and reach top corporate positions, participate in key decision-making bodies, and have a voice in communities. Furthermore, the achievement of gender equality might significantly change firm networks and ecosystems, with important implications for their performance and survival.

Similarly, research at the nexus of (corporate) *governance*, *career progression*, *compensation* and female *empowerment* could yield useful insights–for example discussing how enterprises, institutions and countries are managed and the impact for women and other minorities. Are there specific governance structures that favor diversity and inclusion?

Lastly, we foresee an emerging stream of research pertaining how the spread of the COVID-19 pandemic challenged women, especially in the workforce, by making gender biases more evident.

For our analysis, we considered a set of 15,465 articles downloaded from the Scopus database (which is the largest abstract and citation database of peer-reviewed literature). As we were interested in reviewing business and economics related gender studies, we only considered those papers published in journals listed in the Academic Journal Guide (AJG) 2018 ranking of the Chartered Association of Business Schools (CABS). All the journals listed in this ranking are also indexed by Scopus. Therefore, looking at a single database (i.e., Scopus) should not be considered a limitation of our study. However, future research could consider different databases and inclusion criteria.

With our literature review, we offer researchers a comprehensive map of major gender-related research trends over the past twenty-two years. This can serve as a lens to look to the future, contributing to the achievement of SDG5. Researchers may use our study as a starting point to identify key themes addressed in the literature. In addition, our methodological approach–based on the use of the Semantic Brand Score and its webapp–could support scholars interested in reviewing other areas of research.

## Supporting information

S1 Text Keywords used for paper selection. (PDF)

## References

[1] Amirkhanyan H, Krawczyk MW, Wilamowski M. Gender inequality and national gender gaps in overconfidence. PLOS ONE. 2021;16(4):e0249459 doi: 10.1371/journal.pone.0249459 33857186 PMC8049476

[2] Fisher B, Naidoo R. The geography of gender inequality. PLOS ONE. 2016;11(3):e0145778 doi: 10.1371/journal.pone.0145778 26930356 PMC4773071

[3] Stoet G, Geary DC. A simplified approach to measuring national gender inequality. PLOS ONE. 2019;14(1):e0205349 doi: 10.1371/journal.pone.0205349 30605478 PMC6317789

[4] Maceira HM. Economic benefits of gender equality in the EU. Intereconomics. 2017;52(3):178–83

[5] Minasyan A, Zenker J, Klasen S, Vollmer S. Educational gender gaps and economic growth: A systematic review and meta-regression analysis. World Development. 2019;122:199–217

[6] Esteve-Volart B. Gender discrimination and growth: theory and evidence from India. London School of Economics and Political Science 2004.

[7] Cuberes D, Teignier M. Gender inequality and economic growth: A critical review. Journal of International Development. 2014;26(2):260–76

[8] Abu-Ghaida D, Klasen S. The costs of missing the Millennium Development Goal on gender equity. World Development. 2004;32(7):1075–107

[9] UN. Transforming our world: The 2030 Agenda for Sustainable Development. General Assembley 70 Session; 2015.

[10] Asadikia A, Rajabifard A, Kalantari M. Systematic prioritisation of SDGs: Machine learning approach. World Development. 2020

[11] Nature. Get the Sustainable Development Goals back on track. Nature. 2020;577(January 2):7–8 10.1038/d41586-019-03907-4 31894154

[12] Deressa TT, Hassan RM, Ringler C, Alemu T, Yesuf M. Determinants of farmers’ choice of adaptation methods to climate change in the Nile Basin of Ethiopia. Global Environmental Change. 2009;19(2):248–55

[13] Dumais SA. Cultural capital, gender, and school success: The role of habitus. Sociology of Education. 2002;75(1):44–68

[14] Kamin T, Vezovnik A. Slovenia’s socialist superwoman: feeding the family, nourishing the nation. Faminist Review. 2017;117(1):79–96

[15] Kang M, Park HJ, Park J. Teachers as good mothers, mothers as good teachers: Functional and ideological work–family alignment in the South Korean teaching profession. Gender, Work and Organization. 2020;27(3):395–413

[16] Smith N, Parrotta P. Why so few women on boards of directors? Empirical evidence from danish companies in 1998–2010. Journal of Business Ethics. 2018;147(2):445–67

[17] Smith N, Smith V, Verner M. Why are so few females promoted into CEO and vice president positions? Danish empirical evidence, 1997–2007. ILR Review. 2013;66(2):380–408

[18] Adams RB, Ferreira D. Women in the boardroom and their impact on governance and performance. Journal of Financial Economics. 2009;94(2):291–309

[19] Campbell K, Mínguez-Vera A. Gender diversity in the boardroom and firm financial performance. Journal of Business Ethics. 2008;83(3):435–51

[20] Yazilitas D, Svensson J, de Vries G, Saharso S. Gendered study choice: A literature review. A review of theory and research into the unequal representation of male and female students in mathematics, science, and technology. Educational Research and Evaluation. 2013;19(6):525–45

[21] Bishu SG, Alkadry MG. A systematic review of the gender pay gap and factors that predict it. Administration & Society. 2017;49(1):65–104

[22] Jackson JF, O’Callaghan EM. What do we know about glass ceiling effects? A taxonomy and critical review to inform higher education research. Research in Higher Education. 2009;50(5):460–82

[23] Bark ASH, Escartín J, van Dick R. Gender and leadership in Spain: A systematic review of some key aspects. Sex Roles. 2014;70(11–12):522–37

[24] Prashar S, Vijay TS, Parsad C. Women entrepreneurship in India: a review of barriers and motivational factors. International Journal of Entrepreneurship and Innovation Management. 2018;22(3):206–19

[25] Terjesen S, Sealy R, Singh V. Women directors on corporate boards: A review and research agenda. Corporate Governance: An International Review. 2009;17(3):320–37

[26] Kirsch A. The gender composition of corporate boards: A review and research agenda. The Leadership Quarterly. 2018;29(2):346–64

[27] Köllen T. Diversity management: A critical review and agenda for the future. Journal of Management Inquiry. 2019

[28] Grau SL, Zotos YC. Gender stereotypes in advertising: a review of current research. International Journal of Advertising. 2016;35(5):761–70

[29] Ahuja MK. Women in the information technology profession: A literature review, synthesis and research agenda. European Journal of Information Systems. 2002;11(1):20–34

[30] Rai SM, Brown BD, Ruwanpura KN. SDG 8: Decent work and economic growth-A gendered analysis. World Development. 2019;113:368–80

[31] Tricco AC, Lillie E, Zarin W, O’Brien KK, Colquhoun H, Levac D, et al. PRISMA extension for scoping reviews (PRISMA-ScR): checklist and explanation. Annals of Internal Medicine. 2018;169(7):467–73 doi: 10.7326/M18-0850 30178033

[32] Teso E, Olmedilla M, Martínez-Torres MR, Toral SL. Application of text mining techniques to the analysis of discourse in eWOM communications from a gender perspective. Technological Forecasting and Social Change. 2018;129:131–42

[33] Sun B, Mao H, Yin C. Male and female users’ differences in online technology community based on text mining. Frontiers in Psychology. 2020;11 doi: 10.3389/fpsyg.2020.00806 32528342 PMC7264420

[34] Karami A, White CN, Ford K, Swan S, Spinel MY. Unwanted advances in higher education: Uncovering sexual harassment experiences in academia with text mining. Information Processing & Management. 2020;57(1):102167

[35] Hasan MR, Nasreen M, Chowdhury MA. Gender-inclusive disaster management policy in Bangladesh: A content analysis of national and international regulatory frameworks. International Journal of Disaster Risk Reduction. 2019;41:101324

[36] Fronzetti Colladon A. The Semantic Brand Score. Journal of Business Research. 2018;88:150–60. 10.1016/j.jbusres.2018.03.026

[37] Fronzetti Colladon A, Grippa F. Brand intelligence analytics. In: Przegalinska A, Grippa F, Gloor PA, editors. Digital Transformation of Collaboration. Cham, Switzerland: Springer Nature Switzerland; 2020. p. 125–41. doi: 10.1371/journal.pone.0233276

[38] Munn Z, Peters MDJ, Stern C, Tufanaru C, McArthur A, Aromataris E. Systematic review or scoping review? Guidance for authors when choosing between a systematic or scoping review approach. BMC Medical Research Methodology. 2018;18(143):1–7 30453902 10.1186/s12874-018-0611-x PMC6245623

[39] Griffiths TL, Steyvers M, editors. Finding scientific topics. National academy of Sciences; 2004. 10.1073/pnas.0307752101 PMC387300 14872004

[40] Mimno D, Wallach H, Talley E, Leenders M, McCallum A, editors. Optimizing semantic coherence in topic models. 2011 Conference on Empirical Methods in Natural Language Processing; 2011.

[41] Wang C, Blei DM, editors. Collaborative topic modeling for recommending scientific articles. 17th ACM SIGKDD international conference on Knowledge discovery and data mining 2011.

[42] Arksey H, O’Malley L. Scoping studies: towards a methodological framework. International Journal of Social Research Methodology. 2005;8(1):19–32

[43] Chiarello F, Belingheri P, Bonaccorsi G, Martini M, Fantoni G. Value creation in emerging technologies through text mining: The case of blockchain. Technology Analysis & Strategic Management. 2021

[44] Mazzei D, Chiarello F, Fantoni G. Analyzing social robotics research with natural language processing techniques. Cognitive Computation. 2020

[45] Feinerer I. An introduction to text mining in R. R News. 2008;8:19–22

[46] Straka M, Straková J, editors. Tokenizing, pos tagging, lemmatizing and parsing ud 2.0 with udpipe. CoNLL 2017 Shared Task: Multilingual Parsing from Raw Text to Universal Dependencies; 2017.

[47] Siddiqi S, Sharan A. Keyword and keyphrase extraction techniques: a literature review. Journal of Computer Applications. 2015;109(2)

[48] Newman D, Koilada N, Lau JH, Baldwin T, editors. Bayesian text segmentation for index term identification and keyphrase extraction. COLING 2012; 2012.

[49] Lu Y, Li, R., Wen K, Lu Z, editors. Automatic keyword extraction for scientific literatures using references. 2014 IEEE International Conference on Innovative Design and Manufacturing (ICIDM); 2014.

[50] Yujian L, Bo L. A normalized Levenshtein distance metric. IEEE Transactions on Pattern Analysis and Machine Intelligence. 2007;29(6):1091–5 doi: 10.1109/TPAMI.2007.1078 17431306

[51] Collobert R, Weston J, Bottou L, Karlen M, Kavukcuoglu K, Kuksa P. Natural language processing (almost) from scratch. Journal of Machine Learning Research. 2011;12:493–2537

[52] Singh J, Gupta V. An efficient corpus-based stemmer. Cognitive Computation. 2017;9(5):671–88

[53] Chiarello F, Trivelli L, Bonaccorsi A, Fantoni G. Extracting and mapping industry 4.0 technologies using wikipedia. Computers in Industry. 2018;100:244–57

[54] Trivelli L, Apicella A, Chiarello F, Rana R, Fantoni G, Tarabella A. From precision agriculture to Industry 4.0. British Food Journal. 2019;121(1730–1743)

[55] Roelleke T, Wang J, editors. TF-IDF uncovered. 31st Annual International ACM SIGIR Conference on Research and Development in Information Retrieval—SIGIR ‘08; 2008.

[56] Mihalcea R, Tarau P, editors. TextRank: Bringing order into text. 2004 Conference on Empirical Methods in Natural Language Processing; 2004.

[57] Blei D. Probabilistic topic models. Communications of the ACM. 2012;55(4):77–84

[58] Iannone F, Ambrosino F, Bracco G, De Rosa M, Funel A, Guarnieri G, et al., editors. CRESCO ENEA HPC clusters: A working example of a multifabric GPFS Spectrum Scale layout. 2019 International Conference on High Performance Computing & Simulation (HPCS); 2019.

[59] Keller KL. Conceptualizing, measuring, and managing customer-based brand equity. Journal of Marketing. 1993;57(1):1–22

[60] Wasserman S, Faust K. Social network analysis: Methods and applications: Cambridge University Press; 1994.

[61] Fronzetti Colladon A, Naldi M. Distinctiveness centrality in social networks. PLOS ONE. 2020;15(5):e0233276. doi: 10.1371/journal.pone.0233276 32442196 PMC7244137

[62] Brandes U. A faster algorithm for betweenness centrality. Journal of Mathematical Sociology. 2001;25(2):163–77

[63] Freeman LC. Centrality in social networks conceptual clarification. Social Networks. 1979;1(3):215–39

[64] Blau FD, Kahn LM. The gender wage gap: Extent, trends, and explanations. Journal of Economic Literature. 2017;55(3):789–865

[65] Budig MJ, England P. The wage penalty for motherhood. American Sociological Review. 2001;66(2):204–25

[66] Bertrand M, Hallock KF. The gender gap in top corporate jobs. ILR Review. 2001;55(1):3–21

[67] Segovia‐Pérez M, Castro Núñez RB, Santero Sánchez R, Laguna Sánchez P. Being a woman in an ICT job: an analysis of the gender pay gap and discrimination in Spain. New Technology, Work and Employment. 2020;35(1):20–39

[68] Owen AL, Temesvary J. CEO compensation, pay inequality, and the gender diversity of bank board of directors. Finance Research Letters. 2019;30:276–9

[69] Geiler P, Renneboog L. Are female top managers really paid less? Journal of Corporate Finance. 2015;35:345–69

[70] Gregory‐Smith I, Main BG, O’Reilly CA III. Appointments, pay and performance in UK boardrooms by gender. The Economic Journal. 2014;124(574):F109–F28

[71] Carter ME, Franco F, Gine M. Executive gender pay gaps: The roles of female risk aversion and board representation. Contemporary Accounting Research. 2017;34(2):1232–64

[72] Ahamed MM, Wen J, Gupta N. Does board composition affect the gender pay gap? Economics Letters. 2019;184

[73] Cook A, Ingersoll AR, Glass C. Gender gaps at the top: Does board composition affect executive compensation? Human Relations. 2019;72(8):1292–314

[74] England P, Bearak J, Budig MJ, Hodges MJ. Do highly paid, highly skilled women experience the largest motherhood penalty? American Sociological Review. 2016;81(6):1161–89

[75] Bobbitt-Zeher D. The gender income gap and the role of education. Sociology of Education. 2007;80(1):1–22

[76] Gill AM, Leigh DE. Community college enrollment, college major, and the gender wage gap. ILR Review. 2000;54(1):163–81

[77] Loury LD. The gender earnings gap among college-educated workers. ILR Review. 1997;50(4):580–93

[78] Farkas G, England P, Vicknair K, Kilbourne BS. Cognitive skill, skill demands of jobs, and earnings among young European American, African American, and Mexican American workers. Social Forces. 1997;75(3):913–38

[79] Bradley KJ. The incorporation of women into higher education: Paradoxical outcomes? Sociology of Education. 2000;73(1):1–18

[80] Davies S, Guppy N. Fields of study, college selectivity, and student inequalities in higher education. Social Forces. 1997;75(4):1417–38

[81] Correll SJ, Benard S, Paik I. Getting a job: Is there a motherhood penalty? American Journal of Sociology. 2007;112(5):1297–338

[82] Olivetti C, Petrongolo B. Unequal pay or unequal employment? A cross-country analysis of gender gaps. Journal of Labor Economics. 2008;26(4):621–54

[83] Castilla EJ. Gender, race, and meritocracy in organizational careers. American Journal of Sociology. 2008;113(6):1479–526 10.1086/588738 19044141

[84] Castilla EJ, Benard S. The paradox of meritocracy in organizations. Administrative Science Quarterly. 2010;55(4):543–676

[85] Cohen PN, Huffman ML. Working for the woman? Female managers and the gender wage gap. American Sociological Review. 2007;72(5):681–704

[86] Joshi A, Son J, Roh H. When can women close the gap? A meta-analytic test of sex differences in performance and rewards. Academy of Management Journal. 2015;58(5):1516–45

[87] Dezsö CL, Ross DG, Uribe J. Is there an implicit quota on women in top management? A large-sample statistical analysis. Strategic Management Journal. 2016;37(1):98–115

[88] Bao S, Fainshmidt S, Nair A, Vracheva V. Women in upper echelons of management, tenure and legal risk. British Journal of Management. 2014;25(2):388–405

[89] Perryman AA, Fernando GD, Tripathy A. Do gender differences persist? An examination of gender diversity on firm performance, risk, and executive compensation. Journal of Business Research. 2016;69(2):579–86

[90] Terjesen S, Aguilera RV, Lorenz R. Legislating a woman’s seat on the board: Institutional factors driving gender quotas for boards of directors. Journal of Business Ethics. 2015;128(2):233–51

[91] Terjesen S, Singh V. Female presence on corporate boards: A multi-country study of environmental context. Journal of Business Ethics. 2008;83(1):55–63

[92] Oakley JG. Gender-based barriers to senior management positions: Understanding the scarcity of female CEOs. Journal of Business Ethics. 2000;27(4):321–34

[93] Andersson FW, Johansson D, Karlsson J, Lodefalk M, Poldahl A. Female top management in family firms and non-family firms: Evidence from total population data. International Journal of Entrepreneurship and Small Business. 2018;35(3):303–26

[94] Nekhili M, Gatfaoui H. Are demographic attributes and firm characteristics drivers of gender diversity? Investigating women’s positions on French boards of directors. Journal of Business Ethics. 2013;118(2):227–49

[95] Carter DA, D’Souza F, Simkins BJ, Simpson WG. The gender and ethnic diversity of US boards and board committees and firm financial performance. Corporate Governance: An International Review. 2010;18(5):396–414

[96] Khan WA, Vieito JP. CEO gender and firm performance. Journal of Economics and Business. 2013;67:55–66

[97] Sila V, Gonzalez A, Hagendorff J. Women on board: Does boardroom gender diversity affect firm risk? Journal of Corporate Finance. 2016;36:26–53

[98] Palvia A, Vähämaa E, Vähämaa S. Are female CEOs and chairwomen more conservative and risk averse? Evidence from the banking industry during the financial crisis. Journal of Business Ethics. 2015;131(1):577–94

[99] Boulouta I. Hidden connections: The link between board gender diversity and corporate social performance. Journal of Business Ethics. 2013;113:185–97

[100] Nekhili M, Nagati H, Chtioui T, Nekhili A. Gender-diverse board and the relevance of voluntary CSR reporting. International Review of Financial Analysis. 2017;50:81–100

[101] Quadlin N. The mark of a woman’s record: Gender and academic performance in hiring. American Sociological Review. 2018;83(2):331–60

[102] Baixauli-Soler JS, Lucas-Perez ME, Martin-Ugedo JF, Minguez-Vera A, Sanchez-Marin G. Executive directors’ compensation and monitoring: the influence of gender diversity on Spanish boards. Journal of Business Economics and Management. 2016;17(6):1133–54

[103] Lam KC, McGuinness PB, Vieito JP. CEO gender, executive compensation and firm performance in Chinese‐listed enterprises. Pacific-Basin Finance Journal. 2013;21(1):1136–59

[104] Gupta VK, Mortal SC, Guo X. Revisiting the gender gap in CEO compensation: Replication and extension of Hill, Upadhyay, and Beekun’s (2015) work on CEO gender pay gap. Strategic Management Journal. 2018;39(7):2036–50

[105] Koenig AM, Eagly AH, Mitchell AA, Ristikari T. Are leader stereotypes masculine? A meta-analysis of three research paradigms. Psychological Bulletin. 2011;137(4):616–42 doi: 10.1037/a0023557 21639606

[106] Beaman L, Chattopadhyay R, Duflo E, Pande R, Topalova P. Powerful women: does exposure reduce bias? Quarterly Journal of Economics. 2009;124(4):1497–540

[107] Derks B, Van Laar C, Ellemers N. The queen bee phenomenon: Why women leaders distance themselves from junior women. The Leadership Quarterly. 2016;27(3):456–69

[108] Gupta VK, Turban DB, Wasti SA, Sikdar A. The role of gender stereotypes in perceptions of entrepreneurs and intentions to become an entrepreneur. Entrepreneurship, Theory and Practice. 2009;33(2):397–417

[109] Westhead P, Solesvik MZ. Entrepreneurship education and entrepreneurial intention: Do female students benefit? International Small Business Journal. 2016;34(8):979–1003

[110] Kanze D, Huang L, Conley MA, Higgins ET. We ask men to win and women not to lose: Closing the gender gap in startup funding. Academy of Management Journal. 2018;61(2):586–614

[111] Balachandra L, Briggs T, Eddleston K, Brush C. Don’t pitch like a girl!: how gender stereotypes influence investor decisions. Entrepreneurship Theory and Practice. 2019;43(1):116–37

[112] Marlow S, McAdam M. Gender and entrepreneurship: Advancing debate and challenging myths; exploring the mystery of the under‐performing female entrepreneur. International Journal of Entrepreneurial Behavior & Research. 2013;19(1):114–24

[113] Artz B. Gender and entrepreneurial success: evidence from survey data. Applied Economics Letters. 2017;24(3):163–6

[114] Suspitsyna T. Higher education for economic advancement and engaged citizenship: An analysis of the US Department of Education discourse. Journal of Higher Education. 2012;83(1):49–72

[115] Jacobs JA. Gender inequality and higher education. Annual Review of Sociology. 1996;22(1):153–85

[116] Jewkes R, Levin J, Penn-Kekana L. Risk factors for domestic violence: findings from a South African cross-sectional study. Social Science & Medicine. 2002;55(9):1603–17 doi: 10.1016/s0277-9536(01)00294-5 12297246

[117] Levison D, Moe KS, Knaul FM. Youth education and work in Mexico. World Development. 2001;29(1):167–88

[118] Aterido R, Beck T, Iacovone L. Access to finance in Sub-Saharan Africa: Is there a gender gap? World Development. 2013;47:102–20

[119] Sraboni E, Malapit HJ, Quisumbing AR, Ahmed AU. Women’s empowerment in agriculture: What role for food security in Bangladesh? World Development. 2014;61:11–52

[120] Adams T, Gerber JD, Amacker M. Constraints and opportunities in gender relations: Sugarcane outgrower schemes in Malawi. World Development. 2019;122:282–94

[121] Good C, Aronson J, Inzlicht M. Improving adolescents’ standardized test performance: An intervention to reduce the effects of stereotype threat. Journal of Applied Development Psychology. 2003;24(6):645–62

[122] Rydell RJ, McConnell AR, Beilock SL. Multiple social identities and stereotype threat: imbalance, accessibility, and working memory. Journal of Personality and Social Psychology. 2009;96(5):949–66 doi: 10.1037/a0014846 19379029

[123] Hargittai E. Whose space? Differences among users and non-users of social network sites. Journal of Computer-mediated Communication. 2007;13(1):276–97

[124] Lent RW, Lopez AM Jr, Lopez FG, Sheu HB. Social cognitive career theory and the prediction of interests and choice goals in the computing disciplines. Journal of Vocational Behavior. 2008;73(1):52–62

[125] Miller D, Minichilli A, Corbetta G. Is family leadership always beneficial? Strategic Management Journal. 2013;34(5):553–71

[126] Master A, Cheryan S, Meltzoff AN. Computing whether she belongs: Stereotypes undermine girls’ interest and sense of belonging in computer science. Journal of Educational Psychology. 2016;108(3):424–37

[127] Pitman T, Roberts L, Bennett D, Richardson S. An Australian study of graduate outcomes for disadvantaged students. Journal of Further and Higher Education. 2019;43(1):45–57

[128] Chamarbagwala R. Economic liberalization and wage inequality in India. World Development. 2006;34(12):1997–2015

[129] Bertrand M, Black SE, Jensen S, Lleras-Muney A. Breaking the glass ceiling? The effect of board quotas on female labour market outcomes in Norway. Review of Economics Studies. 2019;86(1):191–238

[130] Azam M, Kingdon GG. Are girls the fairer sex in India? Revisiting intra-household allocation of education expenditure. World Development. 2013;42:143–64

[131] Mutua K, Dimitrov DM. Prediction of school enrolment of children with intellectual disabilities in Kenya: The role of parents’ expectations, beliefs, and education. International Journal of Disability, Development and Education. 2001;48(2):179–91

[132] Liddell C, Barrett L, Henzi P. Parental investment in schooling: Evidence from a subsistence farming community in South Africa. International Journal of Psychology. 2003;38(1):54–63

[133] Yueh L. Parental investment in children’s human capital in urban China. Applied Economics. 2006;38(18):2089–111

[134] García-Aracil A, Winter C. Gender and ethnicity differentials in school attainment and labor market earnings in Ecuador. World Development. 2006;34(2):289–307

[135] Ojeda L, Flores LY. The influence of gender, generation level, parents’ education level, and perceived barriers on the educational aspirations of Mexican American high school students. Career Development Quarterly. 2008;57(1):84–95

[136] Gunderson EA, Ramirez G, Levine SC, Beilock SL. New directions for research on the role of parents and teachers in the development of gender-related math attitudes: Response to commentaries. Sex Roles. 2012;66(3–4):191–6

[137] Sorariutta A, Silvén M. Quality of both parents’ cognitive guidance and quantity of early childhood education: Influences on pre‐mathematical development. British Journal of Educational Psychology. 2018;88(2):192–215 doi: 10.1111/bjep.12217 29574680

[138] Van der Vleuten M, Jaspers E, Maas I, van der Lippe T. Intergenerational transmission of gender segregation: How parents’ occupational field affects gender differences in field of study choices. British Educational Research Journal. 2018;44(2):294–318

[139] Delaney L, Harmon C, Redmond C. Parental education, grade attainment and earnings expectations among university students. Economics of Education Review. 2011;30(6):1136–52

[140] Daymont TN, Andrisani PJ. Job preferences, college major, and the gender gap in earnings. Journal of Human Resources. 1984;19(3):408–28

[141] Williams JE, Best DL. Measuring sex stereotypes: A multination study, Rev: Sage Publications, Inc; 1990.

[142] Correll SJ. Gender and the career choice process: The role of biased self-assessments. American Journal of Sociology. 2001;106(6):1691–730

[143] Kanadlı SB, Torchia M, Gabaldon P. Increasing women’s contribution on board decision making: The importance of chairperson leadership efficacy and board openness. European Management Journal. 2018;36(1):91–104

[144] Correll SJ. Constraints into preferences: Gender, status, and emerging career aspirations. American Sociological Review. 2004;69(1):93–113

[145] Falco LD, Summers JJ. Improving career decision self-efficacy and STEM self-efficacy in high school girls: Evaluation of an intervention. Journal of Career Development. 2019;46(1):62–76

[146] Roper S, Scott JM. Perceived financial barriers and the start-up decision: An econometric analysis of gender differences using GEM data. International Small Business Journal. 2009;27(2):149–71

[147] Hitsch GJ, Hortaçsu A, Ariely D. What makes you click?—Mate preferences in online dating. Quantitative Marketing and Economics. 2010;8(4):393–427

[148] Craig L, Mullan K. How mothers and fathers share childcare: A cross-national time-use comparison. American Sociological Review. 2011;76(6):834–61

[149] Lörz M, Mühleck K. Gender differences in higher education from a life course perspective: transitions and social inequality between enrolment and first post-doc position. Higher Education. 2019;77(3):381–402

[150] Chekima B, Wafa SAWSK, Igau OA, Chekima S, Sondoh SL Jr. Examining green consumerism motivational drivers: does premium price and demographics matter to green purchasing? Journal of Cleaner Production. 2016;112:3436–50

[151] Bolzendahl C, Brooks C. Women’s political representation and welfare state spending in 12 capitalist democracies. Social Forces. 2007;85(4):1509–34

[152] Matsa DA, Miller AR. A female style in corporate leadership? Evidence from quotas. American Economic Journal: Applied Economics. 2013;5(3):136–69

[153] Fernandez‐Feijoo B, Romero S, Ruiz‐Blanco S. Women on boards: do they affect sustainability reporting? Corporate Social Responsibility and Environmental Management. 2014;21(6):351–64

[154] Ho SS, Li AY, Tam K, Zhang F. CEO gender, ethical leadership, and accounting conservatism. Journal of Business Ethics. 2015;127(2):351–70

[155] Francis B, Hasan I, Park JC, Wu Q. Gender differences in financial reporting decision making: Evidence from accounting conservatism. Contemporary Accounting Research. 2015;32(3):1285–318

[156] Cho Y, Park J, Han SJ, Ho Y. A woman CEO? You’d better think twice! Career Development International. 2019;24(1):91–108

[157] Carrasco A, Francoeur C, Labelle R, Laffarga J, Ruiz-Barbadillo E. Appointing women to boards: Is there a cultural bias? Journal of Business Ethics. 2015;129(2):429–44

[158] Price CR. Gender, competition, and managerial decisions. Management Science. 2012;58(1):114–22

[159] Yan W, Schiehll E, Muller-Kahle MI. Human and relational capital behind the structural power of CEOs in Chinese listed firms. Asia Pacific Journal of Management. 2019;36(3):715–43

[160] Lyness KS, Heilman ME. When fit is fundamental: performance evaluations and promotions of upper-level female and male managers. Journal of Applied Psychology. 2006;91(4):777–85 doi: 10.1037/0021-9010.91.4.777 16834505

[161] Heilman ME, Wallen AS, Fuchs D, Tamkins MM. Penalties for success: reactions to women who succeed at male gender-typed tasks. Journal of Applied Psychology. 2004;89(3):416–27 doi: 10.1037/0021-9010.89.3.416 15161402

[162] Wellington S, Kropf MB, Gerkovich PR. What’s holding women back? Harvard Business Review. 2003;81(6):18–

[163] Kalev A. Cracking the glass cages? Restructuring and ascriptive inequality at work. American Journal of Sociology. 2009;114(6):1591–643

[164] Van den Brink M, Benschop Y, Jansen W. Transparency in academic recruitment: a problematic tool for gender equality? Organization Studies. 2010;31(11):1459–83

[165] Milkman KL, Akinola M, Chugh D. What happens before? A field experiment exploring how pay and representation differentially shape bias on the pathway into organizations. Journal of Applied Psychology. 2015;100(6):1678–712 doi: 10.1037/apl0000022 25867167

[166] Hardy JH III, Tey KS, Cyrus-Lai W, Martell RF, Olstad A, Uhlmann EL. Bias in context: Small biases in hiring evaluations have big consequences. Journal of Management. 2020

[167] Koch AJ, D’Mello SD, Sackett PR. A meta-analysis of gender stereotypes and bias in experimental simulations of employment decision making. Journal of Applied Psychology. 2015;100(1):128–61 doi: 10.1037/a0036734 24865576

[168] Haslam SA, Ryan MK. The road to the glass cliff: Differences in the perceived suitability of men and women for leadership positions in succeeding and failing organizations. The Leadership Quarterly. 2008;19(5):530–46

[169] Lee LM, Waddell GR. Diversity and the timing of preference in hiring decisions. Journal of Economic Behavior & Organization. 2021;184:432–59

[170] Cheryan S, Ziegler SA, Montoya AK, Jiang L. Why are some STEM fields more gender balanced than others? Psychological Bulletin. 2017;143(1):1–35 doi: 10.1037/bul0000052 27732018

[171] Stoet G, Geary DC. The gender-equality paradox in science, technology, engineering, and mathematics education. Psychological Science. 2018;29(4):581–93 doi: 10.1177/0956797617741719 29442575

[172] Steele CM, Aronson J. Stereotype threat and the test performance of academically successful African Americans. In: Jencks C, Phillips M, editors. The Black–White test score gap. Washington, DC: Brookings; 1998. p. 401–27

[173] Shapiro JR, Williams AM. The role of stereotype threats in undermining girls’ and women’s performance and interest in STEM fields. Sex Roles. 2012;66(3–4):175–83

[174] Nguyen HHD, Ryan AM. Does stereotype threat affect test performance of minorities and women? A meta-analysis of experimental evidence. Journal of Applied Psychology. 2008;93(6):1314–34 doi: 10.1037/a0012702 19025250

[175] Schmader T. Gender identification moderates stereotype threat effects on women’s math performance. Journal of Experimental Social Psychology. 2002;38(2):194–201

[176] Flore PC, Wicherts JM. Does stereotype threat influence performance of girls in stereotyped domains? A meta-analysis. Journal of School Psychology. 2015;53(1):25–44 doi: 10.1016/j.jsp.2014.10.002 25636259

[177] Lindberg SM, Hyde JS, Petersen JL, Linn MC. New trends in gender and mathematics performance: A meta-analysis. Psychological Bulletin. 2010;136(6):1123–35 doi: 10.1037/a0021276 21038941 PMC3057475

[178] Heilman ME, Okimoto TG. Why are women penalized for success at male tasks? The implied communality deficit. Journal of Applied Psychology. 2007;92(1):81–92 doi: 10.1037/0021-9010.92.1.81 17227153

[179] Burgess D, Borgida E. Who women are, who women should be: Descriptive and prescriptive gender stereotyping in sex discrimination. Psychology, Public Policy, and Law. 1999;5(3):665–92

[180] Eagly AH, Karau SJ. Role congruity theory of prejudice toward female leaders. Psychological Review. 2002;109(3):573–98 doi: 10.1037/0033-295x.109.3.573 12088246

[181] Reguera-Alvarado N, de Fuentes P, Laffarga J. Does board gender diversity influence financial performance? Evidence from Spain. Journal of Business Ethics. 2017;141(2):337–50

[182] Isidro H, Sobral M. The effects of women on corporate boards on firm value, financial performance, and ethical and social compliance. Journal of Business Ethics. 2015;132(1):1–19

[183] McGuinness PB, Vieito JP, Wang M. The role of board gender and foreign ownership in the CSR performance of Chinese listed firms. Journal of Corporate Finance. 2017;42:75–99

[184] Abdullah SN, Ismail KNIK, Nachum L. Does having women on boards create value? The impact of societal perceptions and corporate governance in emerging markets. Strategic Management Journal. 2016;37(3):466–76

[185] Chapple L, Humphrey JE. Does board gender diversity have a financial impact? Evidence using stock portfolio performance. Journal of Business Ethics. 2014;122(4):709–23

[186] Martín‐Ugedo JF, Mínguez‐Vera A, Palma‐Martos L. Female CEOs, returns and risk in Spanish publishing firms. European Management Review. 2018;15(1):111–20

[187] Jadiyappa N, Jyothi P, Sireesha B, Hickman LE. CEO gender, firm performance and agency costs: evidence from India. Journal of Economic Studies. 2019;46(2):482–95

[188] Costa PT Jr, Terracciano A, McCrae RR. Gender differences in personality traits across cultures: robust and surprising findings. Journal of Personality and Social Psychology. 2001;81(2):322–31 doi: 10.1037/0022-3514.81.2.322 11519935

[189] Moss-Racusin CA, Rudman LA. Disruptions in women’s self-promotion: The backlash avoidance model. Psychology of Women Quarterly. 2010;34(2):186–202

[190] Kray LJ, Thompson L, Galinsky A. Battle of the sexes: gender stereotype confirmation and reactance in negotiations. Journal of Personality and Social Psychology. 2001;80(6):942–58 11414376

[191] Rudman LA, Glick P. Prescriptive gender stereotypes and backlash toward agentic women. Journal of Social Issues. 2001;57(4):743–62

[192] Davis LS, Williamson CR. Does individualism promote gender equality? World Development. 2019;123:104627

[193] Brescoll VL, Uhlmann EL. Can an angry woman get ahead? Status conferral, gender, and expression of emotion in the workplace. Psychological Science. 2008;19(3):268–75 doi: 10.1111/j.1467-9280.2008.02079.x 18315800

[194] Jeppesen LB, Lakhani KR. Marginality and problem-solving effectiveness in broadcast search. Organization Science. 2010;21(5):1016–33

[195] Raver JL, Nishii LH. Once, twice, or three times as harmful? Ethnic harassment, gender harassment, and generalized workplace harassment. Journal of Applied Psychology. 2010;95(2):236–54 doi: 10.1037/a0018377 20230066

[196] Cortina LM, Kabat-Farr D, Leskinen EA, Huerta M, Magley VJ. Selective incivility as modern discrimination in organizations: Evidence and impact. Journal of Management. 2013;39(6):1579–605

[197] Roehling MV, Roehling PV, Pichler S. The relationship between body weight and perceived weight-related employment discrimination: The role of sex and race. Journal of Vocational Behavior. 2007;71(2):300–18

[198] Kossek EE, Su R, Wu L. “Opting out” or “pushed out”? Integrating perspectives on women’s career equality for gender inclusion and interventions. Journal of Management. 2017;43(1):228–54

[199] Vinkenburg CJ, Van Engen ML, Eagly AH, Johannesen-Schmidt MC. An exploration of stereotypical beliefs about leadership styles: Is transformational leadership a route to women’s promotion? The Leadership Quarterly. 2011;22(1):10–21

[200] Ceci SJ, Ginther DK, Kahn S, Williams WM. Women in academic science: A changing landscape. Psychological Science in the Public Interest. 2014;15(3):75–141 doi: 10.1177/1529100614541236 26172066

[201] Zafar B. College major choice and the gender gap. Journal of Human Resources. 2013;48(3):545–95

[202] Walton GM, Logel C, Peach JM, Spencer SJ, Zanna MP. Two brief interventions to mitigate a “chilly climate” transform women’s experience, relationships, and achievement in engineering. Journal of Educational Psychology. 2015;107(2):468–85

[203] Miles R. Employment and unemployment in Jordan: The importance of the gender system. World Development. 2002;30(3):413–27

[204] Vicente-Molina MA, Fernández-Sainz A, Izagirre-Olaizola J. Does gender make a difference in pro-environmental behavior? The case of the Basque Country University students. Journal of Cleaner Production. 2018;176:89–98

[205] Braunstein E. Engendering foreign direct investment: Family structure, labor markets and international capital mobility. World Development. 2000;28(7):1157–72

[206] Chen Z, Ge Y, Lai H, Wan C. Globalization and gender wage inequality in China. World Development. 2013;44:256–66

[207] Meece JL, Glienke BB, Burg S. Gender and motivation. Journal of School Psychology. 2006;44(5):351–73

[208] Aguinis H, Ji YH, Joo H. Gender productivity gap among star performers in STEM and other scientific fields. Journal of Applied Psychology. 2018;103(12):1283–306 doi: 10.1037/apl0000331 30024197

[209] Morley L. Lost leaders: Women in the global academy. Higher Education Research & Development. 2014;33(1):114–28

[210] Hakim C. Women, careers, and work-life preferences. British Journal of Guidance & Counselling. 2006;34(3):279–94

[211] Campbell C, MacPhail C. Peer education, gender and the development of critical consciousness: Participatory HIV prevention by South African youth. Social Science & Medicine. 2002;55(2):331–45 12144146 10.1016/s0277-9536(01)00289-1

[212] Bird SR. Unsettling universities’ incongruous, gendered bureaucratic structures: A case‐study approach. Gender, Work and Organization. 2011;18(2):202–30

[213] Dobbin F, Schrage D, Kalev A. Rage against the iron cage: The varied effects of bureaucratic personnel reforms on diversity. Americal Sociological Review. 2015;80(5):1014–44

[214] Brescoll VL. Leading with their hearts? How gender stereotypes of emotion lead to biased evaluations of female leaders. The Leadership Quarterly. 2016;27(3):415–28

[215] Becker J, Ayman R, Korabik K. Discrepancies in self/subordinates’ perceptions of leadership behavior: Leader’s gender, organizational context, and leader’s self-monitoring. Group & Organization Management. 2002;27(2):226–44

[216] Sczesny S, Kuhnen U. Meta-cognition about biological sex and gender-stereotypic physical appearance: Consequences for the assessment of leadership competence. Personality and Social Psychology Bulletin. 2004;30(1):13–21 doi: 10.1177/0146167203258831 15030639

[217] Lucas JW. Status processes and the institutionalization of women as leaders. Americal Sociological Review. 2003;68(3):464–80

[218] Deschaine JE, Schaffer MA. Strengthening the role of public health nurse leaders in policy development. Policy, Politics, & Nursing Practice. 2003;4(4):266–74

[219] Leicht C, de Moura GR, Crisp RJ. Contesting gender stereotypes stimulates generalized fairness in the selection of leaders. The Leadership Quarterly. 2014;25(5):1025–39

[220] Ogbor JO. Mythicizing and reification in entrepreneurial discourse: Ideology-critique of entrepreneurial studies. Journal of Management Studies. 2000;37(5):605–35

[221] Alkhaled S, Berglund K. ‘And now I’m free’: Women’s empowerment and emancipation through entrepreneurship in Saudi Arabiaand Sweden. Entrepreneurship & Regional Development. 2018;30(7):877–900

[222] Danish AY, Smith HL. Female entrepreneurship in Saudi Arabia: Opportunities and challenges. International Journal of Gender and Entrepreneurship. 2012;4(3):216–35

[223] Movahedi R, Yaghoubi-Farani A. Analysis of the barriers and limitations for the development of rural women’s entrepreneurship. International Journal of Entrepreneurship and Small Business. 2012;15(4):469–87

[224] Roomi MA, Harrison P. Behind the veil: Women-only entrepreneurship training in Pakistan. International Journal of Gender and Entrepreneurship. 2010;2(2):150–72

[225] Javadian G, Singh RP. Examining successful Iranian women entrepreneurs: An exploratory study. Gender in Management: An International Journal. 2012;27(3):148–64

[226] Goebel A. Women and sustainability: What kind of theory do we need? Canadian Woman Studies. 2003;23(1):77–84

[227] Lämsä AM, Vehkaperä M, Puttonen T, Pesonen HL. Effect of business education on women and men students’ attitudes on corporate responsibility in society. Journal of Business Ethics. 2008;82(1):45–58

[228] Galbreath J. Are there gender-related influences on corporate sustainability? A study of women on boards of directors. Journal of Management and Organization. 2011;17(1):17–38

[229] Glass C, Cook A, Ingersoll AR. Do women leaders promote sustainability? Analyzing the effect of corporate governance composition on environmental performance. Business Strategy and the Environment. 2016;25(7):495–511

[230] Arora-Jonsson S. Virtue and vulnerability: Discourses on women, gender and climate change. Global Environmental Change. 2011;21(2):744–51

[231] Agarwal B. Conceptualising environmental collective action: Why gender matters. Cambridge Journal of Economics. 2000;24(3):283–310

[232] Edison Stevenson J, Orr E. We interviewed 57 female CEOs to find out how more women can get to the top. Harvard Business Review. 2017

