# Supplementary material for: Twenty years of gender equality research: A scoping review based on a new semantic indicator
Source: PLoS One. 2021 Sep 21;16(9):e0256474. doi: 10.1371/journal.pone.0256474 (PMC8454943; doi:10.1371/journal.pone.0256474)
Supplement: S1 Text — (PDF) [file pone.0256474.s001.pdf]

S1 Text. Keywords used for paper selection.

The sample of relevant articles for our scoping review was identified in two steps. First, we retrieved from the Scopus database all the articles that contained the term "gender" in their title, abstract or keywords, and were published in a journal listed in the AJG 2018 ranking of the CABS, considering the time period from Jan 2000 to May 2021. In this way, we obtained more than 55,000 articles. Then, we reduced this first sample of articles to keep only those in line with the topic of our scoping review. In particular, we retained those papers that mentioned, in their title and/or abstract, both gender-related keywords and keywords referring to bias and equality issues. The keyword selection process is detailed in the paper, while their full list is provided here:

Equality: *biased; biases; bias; unequally; workequality; equalities; unequal; equal; inequalities; equality; inequality; equities; inequitably; equity; inequitable; equitable; inequities; inequity; homecare; homemakers; homemaking; inclusions; inclusivity; inclusive; homeworker; homeworking; overrepresentation; homeworkers; housekeeper; householder; inclusiveness; inclusion; quotas; underrepresented; empower; counterstereotyped; counterstereotyping; metastereotypes; stereotypes; discriminatory; empowering; stereotypical; barriers; empowerment; stereotyped; stereotyping; discrimination; stereotype.*

Gender: *brides; bride; bridal; granddaughters; daughter; daughters; daughterhood; diversities; diverse; diversity; femaling; femaleness; females; female; afeminist; antifeminist; cyberfeminist; feminism; postfeminists; defeminization; feminising; hyperfemininity; cyberfeminism; ecofeminism; feminised; ecofeminist; feminizing; postfeminist; feminized; postfeminism; feminisms; feminisation; femininities; brideprice; bridewealth; feminists; feminization; feminine; femininity; feminism; feminist; degendered; degenderization; disengender; genderblind; genderedness; genderfied; genderfying; genderization; genderized; genderizing; genderland; girlhoods; genderlessness;samegendered; genderbashing; ungendered; genderlect; genderwise; intergender; grandma; degendering; genderism; regendering; genderless; genders; gendering; gendered; gender; homegirls; supergirls; schoolgirl;*

schoolgirls; girl; girls; lady; ladies; housemaids; nanny; maid; maids; maternalism; matriarchal; mommy; mums; mum; maternity; mothering; motherhood; mother; maternal; mothers; actress; bachelorette; barbiegirls; barbies; divas; cinderella; heroines; heroine; whore; chicks; whores; queens; queen; widows; wifestyles; housewife; housewives; wife; wives; femen; chairwomen; madonna; princess; princesses; churchwomen; clergywomen; cyberwoman; employedwomen; farmwomen; gentlewomanly; gowoman; jazzwomen; saleswomen; servicewomen; sister; sisterhood; superwomen; sisters; womæn; womanless; womanomics; businesswoman; policewomen; womanism; congresswomen; womens; superwoman; womanist; sportswomen; businesswomen; womanhood; woman; women.

This second step led to the identification of 15,465 relevant papers.
